# An EAACI “European Survey on Adverse Systemic Reactions in Allergen Immunotherapy (EASSI)”: the methodology

**DOI:** 10.1186/2045-7022-4-22

**Published:** 2014-07-21

**Authors:** Moises A Calderón, Pablo Rodríguez del Río, Carmen Vidal, Jocelyne Just, Oliver Pfaar, Allan Linneberg, Pascal Demoly

**Affiliations:** 1 Section of Allergy and Clinical Immunology, Imperial College London, National Heart and Lung Institute, Royal Brompton Hospital, London, UK; 2 Allergy Section, Hospital Infantil Universitario Niño Jesús, Madrid, Spain; 3 Allergy Department, Complejo Hospitalario Universitario de Santiago, Santiago de Compostela, Spain; 4 Allergology Department, Centre de l’Asthme et des Allergies. Hôpital d’Enfants Armand-Trousseau (APHP)-Sorbonne Universités, UPMC Univ Paris 06, UMR_S 1136, Institut Pierre Louis d’Epidémiologie et de Santé Publique, Equipe EPAR, Paris, F-75013 75571 Cedex 12, France; 5 Center for Rhinology and Allergology, Department of Otorhinolaryngology, Head and Neck Surgery, University Hospital Mannheim, Wiesbaden, Germany; 6 Research Centre for Prevention and Health, the Capital Region of Denmark, Glostrup, Denmark; 7 Department of Clinical Experimental Research, Glostrup University Hospital, Glostrup, Denmark; 8 Department of Clinical Medicine, Faculty of Health and Medical Sciences, University of Copenhagen, Copenhagen, Denmark; 9 Département de Pneumologie et Addictologie, Hôpital Arnaud de Villeneuve, University Hospital of Montpellier, Sorbonne Universités, UPMC Paris 06, UMR-S 1136, IPLESP, Equipe EPAR, 75013 Paris, France

**Keywords:** Allergen, Adverse systemic reactions, Allergen immunotherapy, Subcutaneous, Sublingual

## Abstract

At present, there is no European report on clinically relevant systemic reactions due to the regular use of allergen immunotherapy (AIT), administered either subcutaneously or sublingually (SCIT and SLIT, respectively) outside clinical trials. Using an electronic survey and a “harmonised terminology” according to MedDRA, we aimed to prospectively collect systemic adverse reactions due to AIT from real life clinical settings.

Under the framework of the EAACI, a team of European specialists in AIT, pharmacovigilance, epidemiology and drugs regulation set up a web-based prospective pilot survey to be conducted in three European countries (France, Germany and Spain). A designated “national coordinator” was responsible for following ethics requirements relative to each country and to select at least 30 doctors per country.

Patients were recruited the same day they received their first dose of either SCIT or SLIT. Patient inclusion criteria were: adults and children, with IgE mediated pollen, house dust mite, Alternaria, and/or animal dander respiratory allergies who will initiate AIT.

A list of 31 symptoms terms were extracted from the MedDRA (Medical Dictionary for Regulatory Activities) dictionary to harmonize the reporting of all adverse systemic reactions in this survey.

The SurveyMonkey® online instrument was used by participant doctors to submit information directly to a blinded central database.

Three questionnaires were generated: i) the Doctor Questionnaire, ii) the Patient Questionnaire and iii) the Adverse Reaction Questionnaire. A handbook and a mistake report form were given to each doctor.

In this paper, we describe the methodology followed.

## Introduction

Allergen immunotherapy (AIT) is the hallmark of allergic treatments; its disease course-modifying potential has been proven for allergic rhinoconjunctivitis and asthma [1-4]. Despite the beneficial effect of AIT on the natural history of allergic diseases, the potential for systemic adverse reactions may influence its prescription rate.

The reporting of adverse reactions varies according to the authors and the data collected; therefore, it is very important to differentiate the parameters used to calculate the percentage or ratio of adverse reactions after AIT. The AIT-related systemic reaction rate varies amongst the published reports. For example, in a retrospective review of a multicentre AIT database, Roy et al. [5] identified in 258 patients over a period of 2 years, a systemic reaction (SR) rate of 0.043% of visits and 0.025% of injections. Furthermore, Greenberg et al. [6] reported a 7% patients’ rate of systemic reactions in a multicenter prospective survey which included 628 patients (20,588 injections) over a period of 1 year. More recently, in a surveillance survey from 2008 to 2011, the American Academy of Allergy, Asthma & Immunology and the American College of Allergy, Asthma & Immunology members completed an annual survey of subcutaneous (SCIT)-related systemic reactions [7]. No fatal reactions were directly or indirectly reported. The systemic reaction rates were similar for all 3 years (0.1% of injection visits; 83% of practices), as were severity grades. On average, for all 3 years, there were 7.1 grade 1 (mild reactions, urticaria or upper respiratory tract symptoms), 2.6 grade 2 (moderate reactions, reduction in lung function with or without other organs involvement), and 0.4 grade 3 (severe reactions, life-threatening anaphylaxis) SRs per 10,000 injection visits.

Upon reporting adverse systemic reactions, some clinical and methodological factors should be considered; these are: i) patient characteristics (severity of allergic disease, co-morbidities, risks factors), ii) type of allergen extract (native or chemically modified allergen, aqueous, depot or adjuvanted), iii) the route of administration of AIT (SCIT or sublingual, SLIT), iv) the dose of antigen given during the up-dosing and maintenance phases, v) the schedule used (conventional, cluster or rush) and, vi) the experience of the treating physician in the early identification and treatment of the systemic reaction.

In the late 80s, 26 cases of fatal reactions occurred in the UK due to the administration of SCIT [8]. The main factors involved in these fatal reactions were the non-specialised prescription of AIT by general practitioners and the lack of medical surveillance after the administration of SCIT. In the US, 74 fatal reactions occurred from 1973 to 2001 [9-12]. In a US survey [11], it was estimated that fatal reactions occurred once per 2.5 million injections, with an average of 3.4 deaths per year. In this report there were 20 cases directly reported and 21 cases indirectly reported of fatal reactions to AIT by local physicians.

A prospective multicentre Italian real-life survey assessed the safety of SCIT in 1,738 patients [13]. SRs were graded according to the EAACI recommendations, and were classified as immediate or delayed. Vaccines were prescribed according to guidelines; only standardized depot extracts were used. A total of 60,785 injections were given over a mean SCIT duration of 3 years. Overall, 95 reactions were observed in 57 patients (3.28%), corresponding to 4.7% of the courses and 1.56/1000 injections. Twenty-five patients experienced more than one adverse event. There were 34 grade 2, 60 grade 3 and one grade 4 reactions and no fatality [13].

For SLIT, 11 case reports of anaphylaxis (all non-fatal) have been published [14]. These cases were diagnosed according to the World Allergy Organization criteria [15]. The extremely low incidence of systemic serious adverse reactions in the European experience supports its indication for home administration.

At present, data regarding adverse reactions to AIT is retrieved from different sources: clinical development (clinical trials), post marketing surveillance and pharmacovigilance. There is currently no European report network on adverse reactions (clinically relevant systemic reactions) in daily practice.

In recent systematic reviews and meta-analyses [16-21], different authors have highlighted that there is a wide variety of terms and grading systems that have been used to report adverse reactions to AIT [15,22-24], and this has a limiting effect upon the comparability between reports. Despite recent efforts from international allergy societies in grading reactions [15,25], there is still no harmonisation in the terminology used when reporting systemic adverse reactions due to AIT, either SCIT or SLIT.

Considering all these factors, under the framework of the European Academy of Allergy and Clinical Immunology (EAACI), an electronic pilot survey is conducted to prospectively follow a cohort of patients receiving AIT, either SCIT or SLIT. The aim of the study is to estimate i) the proportion of patients experiencing at least one systemic adverse reaction, ii) the incidence rate of systemic reactions in a real life setting and, iii) the possible risk factors involved in these reactions. For this purpose it was proposed the use of a harmonised terminology to properly report adverse reactions by using well-predefined and clearly stated terms following the MedDRA (Medical Dictionary for Regulatory Activities) classification [26].

In this paper, the methodology developed to perform a European prospective electronic survey on clinically relevant systemic reactions due to AIT, either SCIT or SLIT, administrated in a real clinical setting and using a predefined harmonised terminology based on the MedDRA is described. This pilot survey will precede and form the basis for a future large-scale European survey.

## Methodology

The EAACI Immunotherapy Interest Group (ITIG) sought to perform a prospective longitudinal survey to collect information on systemic adverse reactions of AIT in a real life setting. It was decided that a pilot survey was needed to confirm the usefulness of the questionnaires and the feasibility of using them in real life for future large-scale European surveys.

### Survey team

The survey team was composed of 21 people including a responsible person (Chair of the EAACI ITIG), an international survey coordinator, 3 national coordinators, a survey manager, a survey secretary, a medical survey team (7 members) and a survey expert advisers’ team (6 members).

Three working teams were created: i) for the questionnaires and survey development, ii) for the selection of the harmonized terms from MedDRA to describe adverse reactions and, iii) for data analysis and reporting. All teams included experts in different fields of allergy and AIT: clinicians performing AIT, pharmacovigilance, epidemiology, drugs regulation, and institutional EAACI representatives.

### Objectives of the survey

The main objectives of the survey were i) to collect information of systemic adverse reactions in AIT in real life practice and, ii) to evaluate the use of a harmonised MedDRA-based terminology. Secondary objectives were i) to establish the web support of the survey, ii) to optimise the logistics of the future pan-European survey based on this pilot survey and, iii) to offer an estimation on the sample size needed for a future pan-European survey.

### Design of the survey

The survey is to be carried out in a prospective manner, all patients are recruited on the same day they receive their first dose of a new AIT treatment, either SCIT or SLIT, and are followed up until the last day of the survey with a maximum follow up of 18 months and a minimum of 3 months. Only systemic reactions due to AIT are registered. The survey started the 1st of September of 2012 and was completed in February 2014. To keep a closer control on every variable, this pilot survey is being conducted in several centres simultaneously in three separate European countries (France, Germany and Spain). A designated “national coordinator” is responsible for following specific country ethics requirements and for selecting at least 30 doctors per country.

### Design of questionnaires

The SurveyMonkey® online survey instrument was used [27], allowing participant doctors to store all data collected on a centralised database. All data filed was protected by an enhanced security system (Secure Sockets Layer, SSL, a protocol for encrypting information over the Internet). The questionnaires were done in accordance with the “checklist for reporting results of internet e-surveys, CHERRIES” [28].

The survey used a skip logic pattern, allowing participating doctors to avoid certain sections according to their responses in preceding questions. The questions were presented in a fixed order and most of them were close-ended; however, an optional free text box was supplied in some of the questions to avoid missing unexpected information. Most of the questions were designed to be answered in a compulsory manner. The survey was then beta tested by 10 doctors in centres of different countries. Once the questionnaires and the electronic survey had satisfactory fulfilled all academic and logistical issues, they were emailed by the “Survey Coordinator” to each participant’s survey doctor, named the “Survey Doctor”. The survey was designed as a “closed survey” with an individual study doctor’s code and password controlling access to each questionnaire. Once the questionnaires were completed, respondents were allowed to review and change their answers, but after they were “submitted”, answers could not be modified anymore.

### Blinding

**Figure 1:**
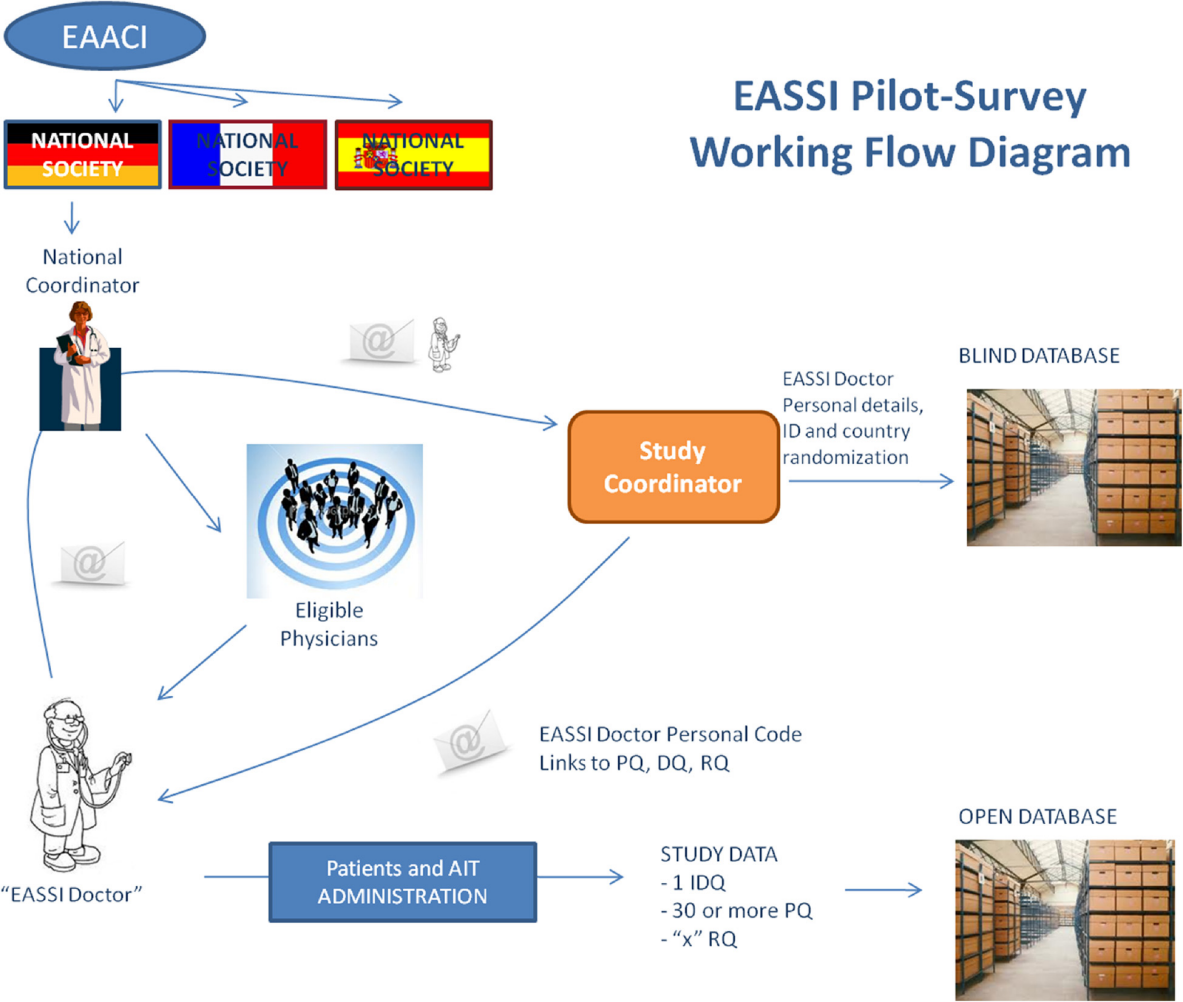
EASSI pilot-survey working flow diagram.

Assuring doctors’ anonymity was crucial for this type of survey, collecting any kind of mistakes and other sensitive information. For this purpose, two separate databases were created. The first database contained names and contact details of participating study doctors, together with an individual numerical identification code for the survey. The second database contained only the survey information extracted from the 3 questionnaires. The Survey International Coordinator is the only person with access to the first database, enabling contact with any study doctor if any clarification regarding patient data was needed. Access to the second database was allowed only to the team responsible for the analysis of the survey data (Figure 1).

## Results

### Questionnaires

Three specific electronic questionnaires were designed. Questions were modified slightly in accordance to ethic committees’ recommendations or requests in each participating country, although no changes in the main body of the survey were required.

The 3 questionnaires generated are: i) Doctor’s Questionnaire (DQ), ii) Patient’s Questionnaire (PQ) and, iii) Adverse Reactions Questionnaire (RQ).

i) **Doctor’s Questionnaire (DQ)** (Additional file 1). This is the first questionnaire to be completed, and the survey doctor should fill it in only once. It is composed of 9 questions; its completion takes 2–3 minutes. The DQ includes information about the doctor prescribing AIT, such as his/her speciality, clinical experience (in years), setting of practice (public or private), percentage of new patients they prescribed to receive AIT in the previous year and the percentage of SCIT or SLIT treatments prescribed in the previous year.

ii) **Patient’s Questionnaire (PQ)** (Additional file 2). This questionnaire should be completed once per each new patient and AIT course included in the survey. It comprises 25 to 34 questions, depending on the options chosen; its completion takes 6–8 minutes. The PQ includes: demographic data of the patient (age, gender), baseline medical history (cardio-vascular problems and other clinically relevant chronic diseases), allergy history (asthma, rhinitis, urticaria, atopic dermatitis, conjunctivitis and food-, drug- or hymenoptera-allergy), any current treatment, patient’s allergic profile (skin prick tests (SPTs), sIgE, clinically relevant), previous AIT (tolerance, composition) and details of the prescribed AIT that will be followed up, along with the survey (onset, composition, route, extract, formulation, schedule, premedication).

iii) **Systemic Adverse Reactions Questionnaire (RQ)** (Additional file 3). This questionnaire was designed to be used only in the event of a systemic adverse reaction occurring in a patient included in this survey. It comprises 17 to 21 questions; its completion takes 4–6 minutes. The data collected includes: treatment phase when the reaction occurred (up-dosing or maintenance), elapsed time from application to onset of systemic adverse reaction, symptoms, treatment used to control the reaction, severity, seriousness, causality, co-factors identified that may have triggered the adverse reaction, duration, and the final outcome of the reaction. It was also asked whether the treatment was discontinued or not; in case it was not discontinued, information was also requested about any modification to the AIT schedule. If a serious adverse event occurs, the survey doctors were advised to forward the information to their national authorities and to follow the reporting procedures established in their countries.

A survey handbook with a brief explanation of every question as well as some key points of the survey was prepared by the medical team and distributed to all participating doctors.

### Minimising data-entry mistakes

In the event that participating survey doctors were aware they had made a mistake entering data, they were advised to complete a Report of Mistakes Form indicating the type of mistake made in order for it to be fixed by the international survey coordinator. A record of all mistakes and changes was kept.

### Data quality assessment

The survey international coordinator and the survey manager carried out a monthly systematic review of the database, searching for inconsistencies. If duplicated patients, missing information or any other error was detected, the survey international coordinator generated a query and contacted the study doctor responsible of that questionnaire for clarification. When a mistake was identified, and after double checking, the mistake was removed and the correct data was introduced into the database. Tracked changes of all errors detected were kept at all times.

### Recruitment

Potential participating survey doctors were recruited by the corresponding national coordinator. Several methods were used such as direct phone calls, emails, open calls and seminars in National and Regional meetings and congresses. Participants were not supported by any fee. Nevertheless, all participant doctors are acknowledged at the end of the paper and those from centres contributing larger numbers of patients are given the option to be named as authors in the publication.Once doctors accepted to participate, they contacted the survey international coordinator who responded with an introductory email containing a formal invitation, the survey protocol, the survey handbook, and 3 uniform resource locators (URLs) to access each questionnaire (Figure 1).

The survey doctor’s inclusion criteria were: to have access to the internet, to have knowledge of English (to understand written English), to administrate AIT (either SCIT or SLIT) as a part of his/her regular clinical practice duties, and to commit to personally collect and transfer the information into the survey database.

### Patient’s in/exclusion criteria

The patient inclusion criteria for the survey were: adults and children, males and females, patients with IgE mediated pollen and/or house dust mite, and/or *Alternaria*, and/or animal dander respiratory allergies who would initiate AIT, either SCIT or SLIT, according to real-life clinical standards of practice. Patients undergoing pre- or co-seasonal treatment might only be included if their first administration of the whole AIT course was during the survey period. Patients could be included in this survey if previous AIT courses had been performed and finished in the past, regardless of the gap between the end of the previous treatment and the start of the new one. Patients undergoing more than one AIT could be included, by completing one PQ for each treatment. Exclusion criteria were: other AIT including venom, moulds other than *Alternaria*, and food immunotherapy.

### Ethics

European Union and specific country ethics, and regulatory requirements, including data protection, were followed. Each survey’s national coordinator was responsible for addressing the corresponding ethics’ committee.

### Harmonised MedDRA terminology

Prior to initiation of the pilot survey, it was decided that all symptoms described as “systemic adverse reactions” will be reported using the MedDRA terms [26]. MedDRA is a clinically validated international medical terminology used to classify adverse events information associated with the use of biopharmaceuticals and other medical products, allowing health authorities and industry to exchange and analyse data related to the safe use of medical products. The use of MedDRA terminology is supported by different international agencies including the European Medicines Agency (EMA).

Since 2003 it has been mandatory to submit all serious adverse events electronically using this dictionary. The MedDRA dictionary is organised by System Organ Class (SOC), divided into High-Level Group Terms (HLGT), High-Level Terms (HLT), Preferred Terms (PT) and finally into Lower-Level Terms (LLT).

After reviewing literature, the medical team selected 31 LLT medical terms from the MedDRA dictionary, to describe the nature of any adverse event elicited during an AIT course (Table 1). Besides these 31 terms, a free text box was provided to gather any unexpected terms that could have been undervalued by the medical team. The included terms are: abdominal pain, angioedema, asthma, blood pressure decrease, bronchospasm, chest discomfort, chest tightness, conjunctivitis allergic, cough, diarrhoea, dysphagia, dysphonia, dyspnoea, dizziness, erythema, fatigue, flushing, generalised erythema, headache, hypotension, laryngeal oedema, loss of consciousness, nausea, generalized pruritus, allergic rhinitis, sensation of foreign body, syncope, tachycardia, urticaria, vomiting and wheezing. Additional descriptions were added to some to avoid misunderstanding for and from the survey doctors. There was no attempt to group them into different severities.

**Table 1:** Selected medical terms from the MedDRA dictionary used in this survey for recording systemic adverse reactions

**SOC**	**PT**	**LLT**
Cardiac disorders	Tachycardia	Tachycardia
Eye disorders	Conjunctivitis allergic	Conjunctivitis allergic
Gastrointestinal disorders	Abdominal pain	Abdominal pain
	Diarrhoea	Diarrhoea
	Dysphagia	Dysphagia
	Nausea	Nausea
	Vomiting	Vomiting
General disorders and administration site conditions	Chest discomfort	Chest discomfort
	Chest discomfort	Chest tightness
	Fatigue	Fatigue
	Sensation of foreign body	Sensation of foreign body
Investigations	Blood pressure decreased	Blood pressure decreased
Nervous system disorders	Dizziness	Dizziness
	Headache	Headache
	Loss of consciousness	Loss of consciousness
	Syncope	Syncope
Respiratory, thoracic and mediastinal disorders	Asthma	Asthma
	Bronchospasm	Bronchospasm
	Cough	Cough
	Dysphonia	Dysphonia
	Dyspnoea	Dyspnoea
	Laryngeal oedema	Laryngeal oedema
	Rhinitis allergic	Rhinitis allergic
	Wheezing	Wheezing
Skin and subcutaneous tissue disorders	Angioedema	Angioedema
	Erythema	Erythema
	Generalised erythema	Generalised erythema
	Pruritus generalised	Pruritus generalized
	Urticaria	Urticaria
Vascular disorders	Flushing	Flushing
	Hypotension	Hypotension

### Statistics

The proportion of patients experiencing systemic reactions during the study period among patients included in the survey will be calculated. 95% confidence intervals (CI) will be based on the binomial distribution. Independent effects of risk factors for systemic effects will be assessed by logistic regression analyses. The observed estimate in this pilot study will be used to properly size the within country/region patient populations in the future large-scale implementation of the survey.

### Data protection

As part of the security measures in the handling and processing of the survey data, it was established that the only authorised person to have access to all information collected by the survey doctors is the survey international coordinator. This person has access to all codes, electronic addresses and personal information of all survey doctors. Regarding patients’ information, there is no chain link to any patient included in the survey. For each patient, an individual national code is created. This code will allow the survey international coordinator to extract any relevant information in case of any systemic adverse reaction.

The SurveyMonkey® facility utilises some of the most advanced technology for Internet security commercially available today. To use this tool, as administrator, a unique user name and password must be entered. SurveyMonkey® issues a session “cookie” only to record encrypted authentication information for the duration of a specific session. Once the user accesses secured areas, the Secure Sockets Layer (SSL) technology protects user information using both server authentication and data encryption, ensuring that user data is safe, secure, and available only to authorized persons. SurveyMonkey® is PCI-DSS compliant.

## Discussion

Based on the academic platform provided by the EAACI, and considering the current lack of information regarding a registry of systemic adverse reactions due to AIT in clinical practice settings across Europe, this electronic survey represents a much-needed and useful tool. The questions included in the three survey questionnaires allow collecting relevant information in a simple, practical, non-commercial and friendly manner.

The general information regarding the survey doctor’s profile requested in the first questionnaire will allow to recognize which factors associated with the AIT prescription are related to doctors themselves. It will be evaluated if specialty (allergy *vs* non-allergy training) and the years of clinical practice may play a role in the selection of patients for AIT and in the early recognition of systemic adverse reactions after AIT and their prompt treatment.

Regarding the patient’s questionnaire, our survey will allow recognition of the factors most commonly linked to systemic adverse reactions. This will be an indirect way of determining risk factors. Attention has been placed upon patients’ medical and allergic history, status of their allergic profile (including other co-allergies), previous successful and non-successful AIT, and previous reactions to AIT. Data on the route of AIT administration, the type of allergens used (native vs allergoid allergens) and the time when reactions occur (build-up vs maintenance phase) will be collected. This survey was designed to collect all possible systemic reactions occurring at AIT clinical practices in real life settings, not as part of clinical trials.

We are aware that total number of doses administered will only give an estimate, and not a specific number since some of the SCIT doses can be administered elsewhere from the doctor’s AIT unit. Patients receiving SLIT were instructed to report any reaction related to treatment which may occur at any time during the study. SLIT doses calculation will be based upon reporting by patients, and not on the count of empty blisters/vials. As mentioned, the incidence rate (number of reactions per patient per month/year of treatment or even better per injection/dose) would probably be more appropriate. Hopefully this may also be calculated, at least as some indirect measure.

It can be argued that adverse events are linked to higher doses of major allergen and thus, some data of efficacy should be provided as a direct sign of good dosage. Again, this survey tries to mirror daily practice and is also aimed to keep it simple, which is why no efficacy parameters are measured.

Only adverse reactions due to AIT for aero-allergens will be recorded, this will provide a more homogeneous treatment modality to be evaluated. For this reason, venom immunotherapy was excluded in this survey. All moulds except for *Alternaria* were also excluded because of the weaker clinical evidence supporting their use in AIT.

Different classifications have been used to record and score adverse reactions due to AIT [15,22-24]. For this survey it was decided to take a different approach; instead of collecting the reactions according to the grade provided by each doctor (which varies considerably), each reaction is described by their single symptoms using the MedDRA classification. This will provide a more detailed description of each reaction, and could also be used to help the future creation of real-life classifications or even allow further collaborations with EMA in the process of registration of any AIT product. The reactions questionnaire was designed to be in accordance with EMA recommendations with respect to the communication of adverse drug reactions

The human team is the key factor in the success of this survey due to their generous collaboration. Despite its reduced budget, this survey has become a reality that will deliver outstanding data concerning one of the most sensitive factors of clinical allergy: systemic adverse reactions resulting from AIT.

## Competing interests

The authors declare that they have no competing interests.

## Authors’ contributions

MC, PRR, CV, JJ, OP, AL and PD have substantially contributed to the conception and design of this project, they drafted and revised it critically for important intellectual content and gave their final approval of the version to be published. All authors read and approved the final manuscript.

## Supplementary Material

Additional file 1 Doctor’s Questionnaire. Click here for file

Additional file 2 Patient’s Questionnaire. Click here for file

Additional file 3 Systemic Adverse Reactions’ Questionnaire. Click here for file
