# Supplementary material for: An EAACI “European Survey on Adverse Systemic Reactions in Allergen Immunotherapy (EASSI)”: the methodology
Source: Clin Transl Allergy. 2014 Jul 21;4:22. doi: 10.1186/2045-7022-4-22 (PMC4113667; doi:10.1186/2045-7022-4-22)
Supplement: Additional file 1 — Doctor’s Questionnaire. [file 2045-7022-4-22-S1.pdf]

Doctor's Questionnaire (DQ)

NOTE: Fill in one D-Q per Doctor, not per center

***1. Personal Doctor Code Identification (assigned by Study Coordinator, information in contact email)**

***2. Which is/are your speciality/ties?**

- [ ] Allergy
- [ ] Pulmonology
- [ ] Dermatology
- [ ] ENT
- [ ] Immunology
- [ ] Pediatrics
- [ ] GP

Others (please specify)

***3. Could you please specify your experience in... ?**

Allergy (in years)

Allergen Specific Immunotherapy (in years)

***4. What is the affiliation of the patients that you will include in this survey?**

- [ ] Public
- [ ] Private

***5. What is the approximate number of NEW patients with respiratory allergy that you see per year?**

***6. Approximately what percentage (%) of these NEW patients go into Allergen Immunotherapy?**

	0	5	10	15	20	25	30	35	40	45	50	55	60	65	70	75	80	85	90	95	100
% of New patients	○	○	○	○	○	○	○	○	○	○	○	○	○	○	○	○	○	○	○	○	○

***7. How are your prescriptions, expressed in percentage (%), divided between both routes?**

Subcutaneous Immunotherapy

Sublingual Immunotherapy

Confirm doctor identification code

Dear doctor, please be so kind to fill in again your identification code in order to minimize data entry mistakes. Thanks for your help.

***8. Personal Doctor Code Identification (assigned by Study Coordinator, information in contact email)**

Doctor's Questionnaire (DQ)

***9. Thank you for completing this questionnaire, do you want to submit it?**

- ○ Yes, when YES is ticked, your questionnaire will be submitted and you will not be able to change any given answer
- ○ No, when NO is ticked, you will be sent to the first question to review the given answers
