# Supplementary material for: An EAACI “European Survey on Adverse Systemic Reactions in Allergen Immunotherapy (EASSI)”: the methodology
Source: Clin Transl Allergy. 2014 Jul 21;4:22. doi: 10.1186/2045-7022-4-22 (PMC4113667; doi:10.1186/2045-7022-4-22)
Supplement: Additional file 2 — Patient’s Questionnaire. [file 2045-7022-4-22-S2.pdf]

Patient's Questionnaire (PQ)

NOTE: Only one P-Q per patient

If you want to enter a patient undergoing 2 or more different SIT treatments, use one patient questionnaire per treatment. Check Handbook v2, p.7

***1. Personal Doctor Code Identification (assigned by Study Coordinator, information in contact email)**

***2. Personal Patient Code Identification (assigned by you at patient´s enrolment)**

***3. Patient´s age at enrolment**

***4. Patient´s gender**

- Female
- Male

***5. Patient´s medical history**

- Cardiovascular disease
- Previous episode of anaphylaxis
- Mastocytosis
- No relevant medical history

Any other relevant disease? please specify:

***6. Patient´s current allergy history**

- Atopic Dermatitis
- Chronic Urticaria
- Asthma
- Drug allergy
- Conjunctivitis
- Food allergy
- Rhinitis
- Hymenoptera allergy

Any other relevant allergic disease? please specify:

***7. Is the patient currently having any active treatment?**

- Yes
- No

Patient's Questionnaire (PQ)

*8. Patient´s use of medication

- ☐ Beta-blockers
- ☐ Inhaled corticosteroids
- ☐ Nasal corticosteroids
- ☐ Long Acting Beta Agonists (LABA)
- ☐ Short Acting Beta Agonists (SABA)
- ☐ Antileukotrienes
- ☐ Anticholinergics
- ☐ Antihistamines
- ☐ Sodium cromoglycate
- ☐ MAO Inhibitors
- ☐ ACE Inhibitors
- ☐ None

Any other drug? please specify:

*9. Patient´s allergy profile: Tick where suitable for sensitisation diagnosis (SPT and/or specific IgE) and if clinically significant

	SPT>3mm	sIgE>0.7kU/L	Clinically significant
Pollen	☐	☐	☐
Mites	☐	☐	☐
Food	☐	☐	☐
Epithelia	☐	☐	☐
Molds	☐	☐	☐
Hymenoptera	☐	☐	☐
Latex	☐	☐	☐

Other (specify)

*10. Is the patient sensitised to pollen?

○ Yes ○ No

11. Indicate which pollens the patient is sensitised to:

- ☐ Grass
- ☐ Birch
- ☐ Olive
- ☐ Ash
- ☐ Plane tree
- ☐ Cupressus
- ☐ Hazel
- ☐ Alder
- ☐ Mugwort
- ☐ Ragweed
- ☐ Parietaria
- ☐ Plantago
- ☐ Chenopodium
- ☐ Saltwort

If other, please specify

*12. Has the patient received any Allergen Specific Immunotherapy (SIT) before?

○ Yes ○ No

Regarding PREVIOUS Allergen SIT, indicate:

Patient's Questionnaire (PQ)

***13. Which route?**

- ○ Sublingual
- ○ Subcutaneous
- ○ Both (in case more than one immunotherapy course)

Regarding PREVIOUS Allergen SIT, indicate:

***14. Its composition:**

- ☐ Grasses
- ☐ Dust Mites
- ☐ Hymenoptera
- ☐ Trees
- ☐ Epithelia
- ☐ Latex
- ☐ Weeds
- ☐ Molds
- ☐ Food

***15. Its tolerance:**

- ○ Very Good (no local, no systemic reactions)
- ○ Good (only local reactions)
- ○ Moderate (local and systemic reactions or systemic reaction only)
- ○ Poor (one or more severe systemic reactions)
- ○ Very Poor (any anaphylactic shock)

Regarding PREVIOUS Allergen SIT, indicate:

***16. For the previous SLIT performed by the patient, indicate its composition:**

- ☐ Grasses
- ☐ Dust Mites
- ☐ Hymenoptera
- ☐ Trees
- ☐ Epithelia
- ☐ Latex
- ☐ Weeds
- ☐ Molds
- ☐ Food

***17. For the previous SLIT performed by the patient, indicate its tolerance:**

- ○ Very Good (no local, no systemic reactions)
- ○ Good (only local reactions)
- ○ Moderate (local and systemic reactions or systemic reaction only)
- ○ Poor (one or more severe systemic reactions)
- ○ Very Poor (any anaphylactic shock)

***18. For the previous SCIT performed by the patient, indicate its composition:**

- ☐ Grasses
- ☐ Dust Mites
- ☐ Hymenoptera
- ☐ Trees
- ☐ Epithelia
- ☐ Latex
- ☐ Weeds
- ☐ Molds
- ☐ Food

Patient's Questionnaire (PQ)

*** 19. For the previous SCIT performed by the patient, indicate its tolerance:**

- Very Good (no local, no systemic reactions)
- Good (only local reactions)
- Moderate (local and systemic reactions or systemic reaction only)
- Poor (one or more severe systemic reactions)
- Very Poor (any anaphylactic shock)

CURRENT Allergen Specific Immunotherapy treatment

*** 20. For the current Allergen SIT, indicate the year of onset of the allergic disease (YYYY)**

*** 21. Date (DD/MM/YYYY) when the first dose of the current Allergen SIT was administered**

*** 22. Composition of current Allergen SIT**

- Grass (or grass mix)
- Tree (or tree mix)
- Mites (or mite mix)
- Alternaria (other molds are excluded)
- Weed
- Epithelia
- Mixture (Non-taxonomically related allergens)

Other (please specify)

Current Allergen Specific Immunotherapy treatment

*** 23. Number of allergens included in current Allergen SIT mixture**

- Two
- Three
- More

*** 24. Who will administrate Allergen SIT in this patient?**

- Physician
- Nurse
- Self administration

Others (please specify)

*** 25. What is the administration route in the current Allergen SIT?**

- Subcutaneous
- Sublingual

Current Allergen Specific Immunotherapy treatment

Patient's Questionnaire (PQ)

***26. Which type of extract?**

- ○ Natural
- ○ Allergoid

Current Allergen Specific Immunotherapy treatment

***27. What type of formulation?**

- ○ Drops
- ○ Tablets

***28. Type of up-dosing schedule**

- ○ Conventional
- ○ Cluster
- ○ Rush

***29. State the frequency of doses given during maintenance per month:**

- ○ Daily
- ○ Each 48 hours
- ○ Weekly
- ○ Every two weeks
- ○ Monthly

***30. Current Allergen SIT is followed to which schedule?**

- ○ Perennial
- ○ Co-seasonal
- ○ Pre-seasonal
- ○ Pre/Co-seasonal

***31. How many months of Allergen SIT is planned to be given in a year?**

[ ]

***32. Use of premedication for current Allergen SIT?**

- ○ Antihistamines
- ○ Corticosteroids
- ○ None

Other (please specify)

[ ]

Confirm doctor and patient identification code

Dear doctor, please be so kind to fill in again your and the patient identification code in order to minimize data entry mistakes.
Thanks for your help.

***33. Personal Doctor Code Identification (assigned by Study Coordinator, information in contact email)**

[ ]

***34. Personal Patient Code Identification (assigned by you at patient´s enrolment)**

[ ]

***35. Thanks for completing this questionnaire, do you want to submit?**

- ○ Yes, when YES is ticked, your questionnaire will be submitted and you will not be able to change any given answer
- ○ No, when NO is ticked, you will be sent to the first question to review the given answers
