# Supplementary material for: An EAACI “European Survey on Adverse Systemic Reactions in Allergen Immunotherapy (EASSI)”: the methodology
Source: Clin Transl Allergy. 2014 Jul 21;4:22. doi: 10.1186/2045-7022-4-22 (PMC4113667; doi:10.1186/2045-7022-4-22)
Supplement: Additional file 3 — Systemic Adverse Reactions’ Questionnaire. [file 2045-7022-4-22-S3.pdf]

Systemic Adverse Reactions' Questionnaire (RQ)

ONLY Adverse Reactions (those events that you consider caused by AIT)

***1. Personal Doctor Code Identification (assigned by Study Coordinator, information in contact email)**

***2. Personal Patient Code Identification (assigned by you at patient´s enrolment)**

***3. Patient´s age at enrolment**

***4. Patient´s gender**

- Female
- Male

***5. At which phase was the Adverse Reaction reported?**

- Up-dosing (increasing dose)
- Maintenance

***6. Indicate the date when the reported adverse reaction was started (DD/MM/YYYY)**

Systemic Adverse Reactions' Questionnaire (RQ)

*7. Type of Adverse Reaction according to the MedDRA Classification for Systemic Reactions:

NOTE 1: ONLY Systemic Adverse Reactions will be recorded (Symptoms at application and/or injection site will NOT be considered)
NOTE 2: According to symptoms reported, further classifications will be done (ie. anaphylactic reaction...)

- ☐ Abdominal pain
- ☐ Angioedema (Deeper swelling of skin/mucosa; single or multiple sites. Could not be well circumscribed & not itchy)
- ☐ Asthma
- ☐ Blood pressure decreased (Suspicion of hypotension, but blood pressure not measured)
- ☐ Bronchospasm
- ☐ Chest discomfort
- ☐ Chest tightness
- ☐ Conjunctivitis allergic (Eye swelling, pruritus, hyperaemia)
- ☐ Cough
- ☐ Diarrhoea
- ☐ Dysphagia (Swallowing difficulty/disorder)
- ☐ Dysphonia (Voice alteration)
- ☐ Dyspnoea
- ☐ Dizziness
- ☐ Erythema (Not at injection/application site but localised abnormal redness of the skin without any raised lesions)
- ☐ Fatigue
- ☐ Flushing (Generalised flushing)
- ☐ Generalised erythema
- ☐ Headache
- ☐ Hypotension (Blood pressure measured Systolic < 90mmHg or > 30% below baseline value)
- ☐ Laryngeal oedema (Objective glottic or vocal cord oedema)
- ☐ Loss of consciousness
- ☐ Nausea
- ☐ Pruritus generalized
- ☐ Rhinitis allergic (Rhinorrhoea, Sneezing, nasal congestion/itching)
- ☐ Sensation of foreign body
- ☐ Syncope (Vasovagal, fainting)
- ☐ Tachycardia (Significant increase of the cardiac rhythm)
- ☐ Urticaria (Generalized/local urticaria. wheals, hives)
- ☐ Vomiting
- ☐ Wheezing

Other, please specify (If this reaction occurred in a patient undergoing 2 or more different SIT treatments, all included in this study, please enter the composition of the extract that has caused the reaction in this free text box)

[ ]

*8. Please record medication used to treat the reaction

- ☐ Adrenaline/ epinephrine intramuscular
- ☐ Adrenaline/ epinephrine intravenous
- ☐ Adrenaline/ epinephrine subcutaneous
- ☐ Antihistamines intramuscular
- ☐ Antihistamines intravenous
- ☐ Antihistamines oral
- ☐ Corticosteroids intramuscular
- ☐ Corticosteroids intravenous
- ☐ Corticosteroids oral
- ☐ Beta 2 agonists
- ☐ Cardiopulmonary resuscitation
- ☐ Fluid (saline)
- ☐ Fluid (expanders)
- ☐ Glucagon
- ☐ Oxygen
- ☐ Transfer to intensive care unit treatment
- ☐ Vasopressin
- ☐ None

If Others, please specify

[ ]

Systemic Adverse Reactions' Questionnaire (RQ)

*9. Severity of reaction

- Mild (Symptoms that don´t interfere with daily activities)
- Moderate (Strong symptoms that interfere regularly in daily activities)
- Severe (Unacceptable symptoms that interfere considerably in daily activities)

*10. Seriousness of reaction

- No
- Yes (Death, life-threatening [risk of death at the time of the event, it does not refer to an event which hypothetically might have caused death if it were more severe], requires in-patient hospitalisation or prolongation of existing hospitalisation, results in persistent or significant disability or incapacity, or a congenital anomaly/birth defect; Medical and scientific judgment considered serious reactions)

*11. Causality of Adverse Reaction (relationship to administration of SIT):

- YES, Possible (causal relationship to SIT is reasonable and cannot be dismissed)
- NO, Unlikely (likely related to another etiology than SIT; i.e. other drugs or underlying disease)

*12. Have you identified any cofactor(s) influencing the Adverse Reaction?

- Exercise
- Anti-inflammatory drugs
- Infection
- Exposure to allergen(s)
- Uncontrolled asthma
- Oral mucosa problem
- None

If Others, please specify

*13. Elapsed time from last Allergen SIT administration to the Systemic Adverse Reaction

Days

Hours

Minutes

*14. The Systemic Adverse Reaction lasted:

Days

Hours

Minutes

*15. Outcome of Systemic Adverse Reaction

*16. After the Adverse Reaction, has the Allergen SIT been discontinued?

- Yes
- No

Systemic Adverse Reactions' Questionnaire (RQ)

***17. Did you modify the schedule?**

- Yes
- No

***18. Which modification did you apply?**

- Same dose repeated
- Reduction >50% of dose in next administration
- Reduction ≤50% of dose in next administration
- Started again

Other (please specify)

Confirm doctor and patient identification code

Dear doctor, please be so kind to fill in again your and the patient identification code in order to minimize data entry mistakes.
Thanks for your help.

***19. Personal Doctor Code Identification (assigned by Study Coordinator, information in contact email)**

***20. Personal Patient Code Identification (assigned by you at patient´s enrolment)**

***21. Thanks for completing this questionnaire, do you want to submit?**

- Yes, when YES is ticked, your questionnaire will be submitted and you will not be able to change any given answer
- No, when NO is ticked, you will be sent to the first question to review the given answers
